# A systematic review of non-antibiotic measures for the prevention of urinary tract infections in pregnancy

**DOI:** 10.1186/s12884-018-1732-2

**Published:** 2018-04-13

**Authors:** Flavia Ghouri, Amelia Hollywood, Kath Ryan

**Affiliations:** School of Pharmacy, University of Reading, PO Box 226, Whiteknights, Reading, RG6 6AP UK

**Keywords:** Systematic review, Non-antibiotic measures, Prevention, Urinary tract infection, Antimicrobial resistance, Pregnancy, Behaviour change

## Abstract

**Background:**

Urinary tract infections (UTIs) are common in pregnancy and account for the highest proportion of primary care antibiotic prescriptions issued to pregnant women in the UK. It is well known that antibiotic use is associated with increased antimicrobial resistance and therefore measures to minimise antibiotic use for UTI prevention have been studied. The efficacy and safety of these measures in pregnancy have not been addressed and therefore the aim of this study was to systematically review the literature to identify and evaluate potential measures to prevent UTIs in pregnant women.

**Methods:**

Ten databases (EMBASE, AMED, BNI, CINAHL, Medline, PubMed, PsycINFO, Cochrane Trials, Scopus and Science Direct) were systematically searched in July 2017 for studies reporting non-antibiotic measures to prevent UTIs in pregnancy. The terms (“urinary tract infection” or UTI or bacteriuria or cystitis) AND (prevention) AND (pregnan*) were used. The quality of the publications was appraised using the Critical Appraisal Skills Programme (CASP) checklists for cohort study, case-control study and randomised controlled trial. The results were synthesised using a textual narrative approach.

**Results:**

Search results yielded 3276 publications and after reviewing titles and removing duplicates, 57 full text articles were assessed for eligibility and eight were included in the review. Five different approaches (hygiene measures, cranberry juice, immunisation, ascorbic acid and Canephron® N) have been identified, all of which are reported to be safe in pregnancy.

**Conclusion:**

The quality of the evidence varied considerably and only hygiene measures were supported by evidence to be recommended in practice. Future work needs to concentrate on strengthening the evidence base through improved design and reporting of studies with a focus on immunisation, ascorbic acid and Canephron® N.

**Electronic supplementary material:**

The online version of this article (10.1186/s12884-018-1732-2) contains supplementary material, which is available to authorized users.

## Background

Urinary tract infections (UTIs) account for the highest proportion of primary care antibiotic prescriptions issued to pregnant women in the UK [1]. Pregnant women have an increased susceptibility to UTIs because of physiological changes. The growing uterus can result in urinary retention which predisposes the woman to infection. In addition, hormonal fluctuations relax the ureteral muscle and cause accumulation of urine in the bladder which also increases the chance of developing a UTI [2].

Treatment of UTIs is recommended in pregnancy if bacteria are detected in the urine even if there are no accompanying symptoms i.e. in asymptomatic bacteriuria (ASB) [3]. Both ASB and symptomatic UTIs in pregnancy are risk factors for the development of pyelonephritis which can result in severe maternal morbidity [4]. It is estimated that 20–30% of women with bacteriuria in the first trimester go on to develop pyelonephritis in later trimesters [5]. Therefore, although ASB on its own is not treated in the general population, guidelines published by the European Urological Association (EAU) [3] and Scottish Intercollegiate Guidelines Network (SIGN) [6] recommend screening and treating bacteriuria with or without symptoms. The current management strategy according to these guidelines is to use a short course of antibiotics.

Whilst antibiotics are vital in eradication of UTIs, antimicrobial resistance due to their use is a global health threat [7, 8]. Antimicrobial resistance means that bacteria can survive antibiotic treatment and cause serious or life threatening infections. Use of antibiotics is strongly associated with increasing emergence of resistant bacteria and subsequent redundancy of antibiotics i.e. previously effective antibiotics are losing their efficacy [8, 9]. Unlike the general population, the choice of safe antibiotics in pregnancy is limited because of teratogenic potential e.g. quinolones should be avoided in pregnancy because of a risk of joint malformations in the foetus. Therefore antibiotics becoming ineffective due to antimicrobial resistance is a particular concern in pregnancy as it further limits the range of drugs available to treat infections safely [10]. An example of this in practice is the replacement of trimethoprim with nitrofurantoin as the first line antibiotic to treat UTIs [11] because of an increase in resistance due to its widespread use in the UK [12]. Use of antibiotics can also result in carriage of resistant bacteria by individuals for a period of several months to a year after completing a course of antibiotics [13]. The resistant bacteria can transfer to close physical contacts and may colonise and infect subsequent hosts. This is especially of concern in pregnancy as women can pass on resistant bacteria to the neonate during birth, which is when they are most vulnerable to infection. An example of resistance specific to obstetric practice is the increase in ampicillin resistant neonatal infections due to maternal use of ampicillin [14, 15].

As well as contributing to antimicrobial resistance, antibiotic use in pregnancy also carries the risk of being harmful to the foetus. Recently, a study has found a link between antibiotic use and increased risk of spontaneous miscarriages [16]. Another study assessing the effects of nitrofurantoin, trimethoprim-sulfamethoxazole and cephalosporins which are used to treat UTIS, found an increased risk of birth defects such as oral clefts, oesophageal and anorectal abnormalities in the offspring [17]. In addition, research has also found an association with antibiotic use in pregnancy and functional impairment in children later on in life [18].

In light of the risks, it is essential that the use of antibiotics in pregnancy is carefully considered with a balance struck between the risks and benefits of these drugs. The UK’s 5 year antimicrobial resistance strategy [19] developed by the Department of Health (DH) and Department for Environment Food and Rural Affairs (Defra) identifies seven key areas where action is needed to tackle antimicrobial resistance. One of these key areas is ‘improving infection prevention and control practices’ which will lead to a reduction in the use of antibiotics as infection rates will be minimised. Improving infection prevention is also one of the main recommendations of ‘The Review on Antimicrobial Resistance’ (2016), chaired by economist Jim O’Neill [8]. Non-antibiotic measures to minimise antibiotic use for UTI prevention have been studied but the efficacy and safety of these measures in pregnancy have not been addressed [20]. Therefore, the aim of this systematic review is to identify alternate measures reported in scientific literature which may be used to prevent UTIs in pregnancy. The benefits of non-antibiotic measures to prevent UTIs in pregnancy are two-fold. Firstly, the reduced use of antibiotics will mean that they remain effective for longer, and secondly, medication which is potentially harmful in pregnancy can be avoided.

## Methods

Ten databases (EMBASE, AMED, BNI, CINAHL, Medline, PubMed, PycINFO, Cochrane Trials, Scopus and Science Direct) were searched and the final search string was conducted in July 2017. The inclusion criteria according to PICOS (see Table 1) consisted of studies reporting non-antibiotic measures for the prevention of UTIs in pregnant women.

**Table 1:** Inclusion criteria (PICOS)

Population	Pregnant Women
Intervention	Non-antibiotic prevention measures
Comparator	Any e.g. a placebo
Outcome	Incidence of bacteriuria or UTI
Study Design	Any e.g. randomised control trial (RCT) or observational study

Studies conducted exclusively in non-pregnant groups or in conditions such as diabetes or spinal cord injury were excluded. Search terms were; **P**: (pregnan*), **I**: (prevention or control or management), **O**: (“urinary tract infection” or UTI or bacteriuria or cystitis) as shown in Table 2.

**Table 2:** Search strategy

Database	Search terms	Results
EMBASE	(“urinary tract infection” or UTI or bacteriuria or cystitis) AND (prevention or control or management) AND pregnan*	744
AMED	(“urinary tract infection” or UTI or bacteriuria or cystitis) AND (prevention or control or management) AND pregnan*	0
BNI	(“urinary tract infection” or UTI or bacteriuria or cystitis) AND (prevention or control or management) AND pregnan*	10
CINAHL	(“urinary tract infection” or UTI or bacteriuria or cystitis) AND (prevention or control or management) AND pregnan*	66
Medline	(“urinary tract infection” or UTI or bacteriuria or cystitis) AND (prevention or control or management) AND pregnan*	397
PubMed	(“urinary tract infection” or UTI or bacteriuria or cystitis) AND (prevention or control or management) AND pregnan*	942
PsycINFO	(“urinary tract infection” or UTI or bacteriuria or cystitis) AND (prevention or control or management) AND pregnan*	4
Cochrane Trials	(“urinary tract infection” or UTI or bacteriuria or cystitis) AND (prevention or control or management) AND pregnan*	102
SCOPUS	(TITLE-ABS-KEY (“urinary tract infection” OR UTI OR bacteriuria OR cystitis) AND TITLE-ABS-KEY (prevention or control or management) AND TITLE-ABS-KEY (pregnan*) AND NOT TITLE-ABS-KEY (catheter OR catheter AND associated) AND NOT TITLE-ABS-KEY (antibacterial* OR antibiotic* OR antimicrobial*) Note: additional terms searched using ‘NOT’ due to too many results	1008
ScienceDirect	(“urinary tract infection” or UTI or bacteriuria or cystitis) AND (prevention or control or management) AND pregnan*	3
Manual search		0
Total		3276

The search terms ‘control’ or ‘management’ were initially used but these terms did not yield relevant results therefore this paper focuses on prevention only. The final search strategy is available in Additional file 1.

A manual search of references from included studies was also conducted. The quality of the publications was appraised using the Critical Appraisal Skills Programme (CASP) checklists for cohort study, case-control study and randomised controlled trial [21–23]. The results were analysed and discussed using a narrative synthesis approach.

## Results

**Fig. 1:**
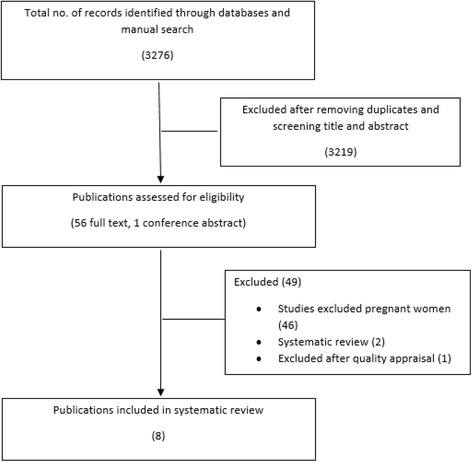
Identification of publications

Search results yielded 3276 publications and after reviewing titles and removing duplicates, 56 full text articles and one conference abstract were assessed for eligibility by FG and eight were included in the review as shown in Fig. 1. The results identified five different measures (hygiene behaviour, cranberry juice, immunisation, ascorbic acid and Canephron® N) which can be used for the prevention of UTIs in pregnancy. Quality appraisal of the included publications using the CASP checklists is shown in Tables 3, Table 4 and Table 5. The characteristics of the publications are included in Table 6.

**Table 3:** Quality appraisal using CASP checklist for cohort studies

CASP cohort study checklist			
	Elzayat et al. 2017 [26]	Baertschi et al. 2003 [29]	Ordzhonikidze et al. 2009 [32]
Did the study address a clearly focused issue?	Yes	Yes	Yes
Was the cohort recruited in an acceptable way?	Yes	Yes	Can’t tell
Was the exposure accurately measured to minimise bias?	Yes	Yes	Can’t tell
Was the outcome accurately measured to minimise bias?	Yes	Yes	Can’t tell
(a) Have the authors identified all important confounding factors?	Yes	Yes	Yes
(b) Have they taken account of the confounding factors in the design and/or analysis?	Yes	Yes	Yes
(a) Was the follow up of subjects complete enough?	Not applicable	Yes	Yes
(b) Was the follow up of subjects long enough?	Not applicable	Yes	Yes
How precise are the results?	Can’t tell (no CI given)	Can’t tell (no CI given)	Can’t tell (no CI given)
Do you believe the results?	Yes	Yes	Yes
Can the results be applied to the local population?	Yes	Yes	No (study population not clearly defined)
Do the results of this study fit with other available evidence?	Yes	Yes	Yes
Does the study have implications for practice?	Yes	Yes	Yes

*CI* Confidence interval. Significance: *p* ≤ 0.05

**Table 4:** Quality appraisal using CASP checklist for case-control studies

CASP case-control study checklist	
	Amiri et al. 2009 [25]
Did the study address a clearly focused issue?	Yes
Did the authors use an appropriate method to answer their question?	Yes
Were the cases recruited in an acceptable way?	Yes
Were the controls selected in an acceptable way?	Yes
Was the exposure accurately measured to minimise bias?	Yes (but questionnaire completed by midwives)
Have the authors taken account of the potential confounding factors in the design and/or in their analysis?	Yes
Were the results and risk estimate precise?	Yes
Do you believe the results?	Yes
Can the results be applied to the local population?	Yes
Do the results of this study fit with other available evidence?	Yes

**Table 5:** Quality appraisal using CASP checklist for randomised controlled trials

CASP randomised control study checklist				
	Ochoa-Brust et al. 2007 [31]	Grischke et al. 1987 [30]	Wing et al. 2008 [27]	Essadi et al. 2010 [28]
Did the trial address a clearly focused issue?	Yes	Yes	Yes	Yes
Was the assignment of patients to treatments randomised?	Yes	N (although described as randomised)	Yes	Can’t tell
Were all of the patients who entered the trial properly accounted for at its conclusion?	No	No	Yes	Yes
Were patients, health workers and study personnel ‘blind’ to treatment?	No	No (only patients were blinded)	Yes	No (able to differentiate between juice and water)
Were the groups similar at the start of the trial?	Yes	No (different pregnancy status)	Yes	Yes
Aside from the experimental intervention, were the groups treated equally?	Yes	Yes	Yes	Can’t tell
How large was the treatment effect?	Significant (*p* = 0.03)	Significant (*p* ≤ 0.001)	Not significant (*p* = 0.71)	Significant (*p* < 0.05)
How precise was the estimate of the treatment effect?	Precise (95% CI used)	Can’t tell (no CI limits)	Precise (95% CI used)	Can’t tell (no CI limits)
Can the results be applied in your context? (Or to the local population)	Yes	Probable	Yes	Yes
Were all clinically important outcomes considered?	Yes	Yes	Yes	Yes
Are the benefits worth the harms and costs?	Yes	Yes	N (due to stomach disturbances)	No (due to stomach disturbances)

*CI* Confidence interval. Significance: *p* ≤ 0.05

**Table 6:** Characteristics of included publications

Author, Year, Country	Wing et al., 2008, USA [28]
Design	Pilot randomised control trial comparing cranberry juice with placebo. Participants were divided into three groups and asked to drink 240 ml of either cranberry or placebo juice. A. cranberry juice three times daily B. cranberry juice once and placebo twice daily C. placebo three times daily Note: High withdrawal led to modification of dose frequency to twice daily in the middle of the trial. Randomisation was stratified by site.
Aim	To determine effectiveness of cranberry juice at reducing the frequency of ASB.
Participants	188 pregnant women < 16 weeks gestation
Key findings	Results report a 57% reduction in bacteriuria and 41% reduction in all UTIs. Authors concluded that cranberries provide protection against ASB as well as symptomatic infections.
Limitations	Small sample size as it was a pilot. About 39% participants dropped out due to gastrointestinal issues.
Author, Year, Country	Essadi et al., 2010, Libya [29]
Design	Randomised control trial comparing cranberry juice to placebo (water). Participants were divided into two groups and asked to drink 250 ml of cranberry juice or water. A: cranberry juice four times daily B: water four times daily Note: This publication is from a conference poster and full details were not available.
Aim	To determine the effectiveness of cranberry juice at reducing the frequency of UTIs.
Participants	760 pregnant women
Key findings	Results report that 70.5% of patients who drank cranberry juice showed a significant reduction (*p* < 0.05) in frequency of UTI compared to 32.16% who drank water. Of women who developed symptomatic UTI, 4.12% delivered prematurely. Authors concluded that cranberry juice has a protective effect in UTI prevention.
Limitations	There was no blinding as cranberry juice is distinguishable from water. High withdrawal rate of participants (28%) attributed to gastrointestinal upset. It is not clear whether authors used intention to treat analysis which may distort results in favour of cranberry juice.
Author, Year, Country	Elzayat et al., 2017, Egypt [26]
Design	An observational study to determine prevalence of ASB and the risk factors associated with it in pregnancy. Urine specimens were collected and analysed to determine ASB. A survey was conducted using a pre-tested questionnaire to gather data for the associated risk factors.
Aim	To determine the prevalence of ASB and identify risk factors associated with it in terms of socioeconomic status or personal hygiene.
Participants	170 pregnant women between the ages of 18–41.
Key findings	The prevalence of ASB was 10% (CI 95% 5.93% to 15.53%) in this sample of pregnant women. There was an association between sexual activity and incidence of ASB and 14% of women with ASB reported sexual activity > twice per week (*p* = 0.01). There was also an association between direction of wiping and 15% of women with ASB reported wiping their genitals from back to front (*p* = 0.03). No other significant association was found. Authors recommended educating women on the significance of personal hygiene to prevent UTI during pregnancy.
Limitations	This is an observational study and data was collected by questionnaire which is subject to accurate participant recall. Confidence intervals were not reported for all the categories.
Author, Year, Country	Amiri et al., 2009, Iran [25]
Design	An observational case-control study. Cases (women with UTI) and controls (no UTI) were matched and compared in terms of difference in genital hygiene or sexual activity. The women were administered a questionnaire by a midwife following which a urine sample was taken for analysis.
Aim	To determine association of genital hygiene and sexual activity with the frequency of UTIs in pregnant women.
Participants	250 pregnant women (100 cases and 150 controls)
Key findings	The authors investigated multiple factors. Of note is the significant association seen with: Sexual activity > thrice a week (OR = 5.62 95% CI: 3.10–10.10) Not voiding the bladder after intercourse (OR = 8.62 95% CI: 6.66–16.66) Washing genital area from back to front (OR - 2.96 95% CI: 1.66–5.28)
Limitations	This was an observational study and data was collected using a questionnaire which is subject to accurate participant recall. Matching of cases and controls is not reported in detail.
Author, Year, Country	Baertschi et al., 2003, Switzerland [33]
Design	A before and after study testing a bacterial extract’s (OM-8930) efficacy and safety in preventing the incidence of UTIs during pregnancy.
Aim	To determine the effect of immunisation on the number of UTI recurrences, the number and duration of antibiotic treatment used and establish the safety of the vaccine (in women or new born).
Participants	62 women 16–28 weeks pregnant
Key findings	The extract significantly reduced the recurrence of UTIs from 52.5% to 19.4% (*p* = 0.002). Number of people needing antibiotic treatment reduced from 55.7% to 12.9% (*p* = 0.0002) Duration of antibiotic treatment reduced from a mean of 3.2 to 2 days (*p* = 0.0016) The authors concluded that OM-8930 reduced the number of UTI recurrences but a larger trial was needed to confirm this result.
Limitations	The study compares data from the trial to the 6 month period prior to the study instead of comparison with a control group. There is a risk of bias due to this because women’s pregnancy status would likely be different at the two times. Also, The study was a pilot and had a small sample size.
Author, Year, Country	Grischke & Ruttgers, 1987, Germany [35]
Design	An open comparative randomised trial comparing effectiveness of a vaccine preparation, Solco-Urovac®, to standard antibiotic therapy for prevention of UTIs. The participants were divided into two groups Group 1: 200 participants given Solco-Urovac® (68 were pregnant) Group 2: 198 participants given nitrofurantoin or another appropriate antibiotic
Aim	To establish the effectiveness of Solco-Urovac® in reducing the frequency of UTIs.
Participants	400 pregnant and non-pregnant women
Key findings	There were 28 infections in the trial group and 84 infections in the control group – this was a significant difference (*p* ≤ 0.001). Average duration of the infection was significantly longer than in the control group. No adverse effects were observed in the offspring.
Limitations	The study was not conducted exclusively in pregnant women and their proportion in each group is not specified. Randomisation was not done appropriately as the treating physician may have allocated patients with acute symptoms to the antibiotic group.
Author, Year, Country	Ochoa-Brust et al., 2007, Mexico [36]
Design	A randomised trial to assess the prophylactic role of ascorbic acid in preventing UTIs during pregnancy. Participants were divided into two groups. Group A: treatment with ferrous sulphate 200 mg, folic acid 5 mg and ascorbic acid 100 mg daily for 3 months Group B: treatment with ferrous sulphate 200 mg and folic acid 5 mg daily for 3 months.
Aim	To determine the role of ascorbic acid in reducing the frequency of UTIs.
Participants	110 pregnant women, 55 in each group.
Key findings	The infection percentage was 12.7% in Group A and 29.1% in Group B (*p* = 0.03, OR 0.35, CI 95% 0.13–0.91). The relative risk reduction was 56.5% and absolute risk reduction was 16.3%, The number needed to treat was 6. The authors concluded that pregnant women in areas with high rates of antimicrobial resistance should take ascorbic acid during gestation to prevent UTIs.
Limitations	Patients were excluded from study if they were not compliant, had serious side effects or if they had a UTI recurrence which may have distorted the results in favour of ascorbic acid.
Author, Year, Country	Ordzhonikidze et al., 2009, Russia [38]
Design	Two groups of pregnant women were treated with Canephron® N. Group 1: 160 women with an exacerbation of pyelonephritis were given Canephron® N in combination with standard therapy (antibiotics). Group 2: 140 women with chronic history of urinary tract disease who were given Canphron® N alone for prevention. The dose of Canephron® N was two tablets three times a day.
Aim	To assess the role of Canephron® N in the management of urinary tract diseases in pregnant women.
Participants	300 pregnant women
Key findings	Group 2 seemed to show more favourable results compared to Group 1. The percentage frequency of exacerbation of pyelonephritis was 10–6.25 in Group 1 and 3–2.1 in Group 2. The authors state in the results section that there was a 1.5-fold decrease in the frequency of infectious complications in the first group and a 1.3-fold decrease in the second group when comparing results to previous years.
Limitations	The methods, results and analysis have not been reported clearly. Canephron® N was not compared to a placebo or to antibiotics.

### Hygiene behaviour

Three observational studies were identified which investigated the association between sexual and genital hygiene behaviours of pregnant women and the incidence of asymptomatic bacteriuria (defined as > 10^5^ colony forming units/ml of urine) or symptomatic UTIs. One study by Badran et al. [24] was not included in the review due to repetition of results from a previously conducted study.

The study by Amiri et al. [25] was a case-control study which included 100 cases matched to 150 controls i.e. total of 250 pregnant women. The two groups were compared in terms of differences in genital hygiene or sexual activity. The study by Elzayat et al. [26] was an observational cohort study that included 170 pregnant women between the ages of 18–41. Participants in this study were administered a questionnaire about their hygiene behaviours and a urine sample was tested to determine the prevalence of bacteriuria. Both studies show that hygiene behaviours are associated with the incidence of UTIs.

### Cranberry juice

There were two studies that assessed the effectiveness of cranberry juice in preventing UTIs during pregnancy. Wing et al. [27] conducted a randomised controlled trial with 188 pregnant women under 16 weeks of pregnancy and compared the efficacy of cranberry juice with a placebo. There was a 57% reduction in bacteriuria and 41% reduction in all UTIs reported in this trial. Essadi et al. [28] conducted a randomised controlled trial that compared cranberry juice with water in 760 pregnant women. They also reported positive results for the effectiveness of cranberry juice and 70.5% of the participants who drank cranberry juice showed a significant reduction in UTIs compared to 32.16% of women who drank water.

### Immunisation

Immunisation as a means of preventing UTIs in pregnancy was assessed by two studies. Baertschi et al. [29] conducted a before-after study using a bacterial extract in 62 women who were 16–28 weeks pregnant. Use of the extract significantly reduced the incidence of UTIs and recurrence rates fell from 52.5% prior to using the extract to 19.4% after women started using the extract. Grischke and Ruttgers [30] investigated the effectiveness of an intramuscular vaccine in an open randomised trial. A total of 400 women were included in the trial and a significant difference was seen in the incidence of UTIs in the trial (28 infections) and control groups (84 infections) suggesting a beneficial effect of the vaccine.

### Ascorbic acid

Ochoa-Brust et al. [31] conducted a RCT to evaluate whether daily intake of ascorbic acid (100 mg) prevented UTIs in pregnancy. There was a total of 110 pregnant women, 55 in the trial group and 55 in the control group. The infection percentage was 12.7% in women who were given daily ascorbic acid compared with 29.1% in women who received the comparator.

### Canephron® N

Ordzhonikidze et al. [32] conducted a cohort study in 300 pregnant women using Canephron® N which is a herbal product. Women were divided into two groups, those who had a current UTI and those who suffered with chronic urinary tract problems but did not have a current exacerbation. The results show that the frequency of pyelonephritis was 1.5 times less in the first group and 1.3 times less in the second group due to use of this product.

## Discussion

The five different measures (hygiene behaviour, cranberry juice, immunisation, ascorbic acid and Canephron® N) highlighted in the review vary in the evidence supporting their use for the prevention of UTIs in pregnancy.

### Hygiene behaviour

The EAU guideline for urological infections states that studies investigating hygiene behaviours have not found any association with the incidence of UTIs [3]. The two observational studies included in this review, however, provide evidence that hygiene behaviours are associated with the incidence of UTIs. Results show that increased sexual activity of greater than two or three times a week was linked to a high frequency of UTIs. However, washing the genital area and voiding the bladder after intercourse had a protective effect. The direction of wiping the genital area after voiding the bladder was also found to be important and women who wiped from back to front had a higher incidence of UTIs according to both studies. Lastly, Amiri et al. [25] also found that drinking inadequate amounts of fluid and delaying voiding of the bladder also increased the likelihood of UTIs.

The overall evidence from these studies supports the adoption of protective hygiene behaviours, which may seem intuitive, as good hygiene is well known to protect against all types of infections. Women should be provided with specific recommendations because they may get upset if they get advised to ‘just keep clean’ as evidenced by a qualitative study conducted by Flower et al. [33].

### Cranberry juice

Both RCTs [27, 28] assessing the efficacy of cranberry juice to prevent UTIs in pregnancy concluded that it has the potential to be effective. However, both studies had limitations which shed doubt on the effectiveness of this intervention. The study by Wing et al. [27] was underpowered with a small sample size (188 women). Essadi et al. [28] had a larger cohort (760 women) but compared cranberry juice to water which led to inadequate blinding giving rise to a risk of performance bias i.e. systemic differences between the groups. In addition, it is not clear if they used intention-to-treat analysis which may have distorted the results in favour of cranberry juice. A point to note with regards to Essadi et al. [28] is that it was published as a conference poster and full details were not available but it was included because the abstract reported data in sufficient detail to determine the significance of the results.

A limitation of cranberry juice seen in both studies was the high volume of juice that needed to be ingested (240 ml [27] and 250 ml [28]). Both trials had a high withdrawal rate mostly due to gastrointestinal disturbances which can limit its use on grounds of acceptability to women. These results point to a need to investigate a standardised content of cranberries in alternative formulations such as tablets and capsules which may help with improving adherence and tolerability of this intervention.

Both these trials view cranberry juice as potentially effective at preventing UTIs in pregnancy but a Cochrane review by Jepson et al. [34] included both these studies in a meta-analysis and found cranberries to be ineffective in preventing UTIs in pregnancy. Thus, although there has been interest in using cranberries for UTI prevention, the evidence does not support its efficacy. It can still be used as a self-care option, if preferred by women, because of its known safety in pregnancy [35, 36].

### Immunisation

Both studies investigating the role of immunisation to safely reduce the recurrence of UTIs in pregnancy found favourable results, however both had significant limitations. Baertschi et al. [29] used a bacterial extract consisting of different strains of *Escherichia coli (E.coli),* which is the most common uropathogen [37], however this vaccine would not be effective against any other type of bacteria. Furthermore it was an open pilot study and did not have a control group to compare the effectiveness of the vaccine. Therefore, the results need to be confirmed by a RCT, as noted by the authors themselves. Grischke and Ruttgers [30] conducted their study in a sample where 68 pregnant women were given the intramuscular vaccine but the number of pregnant women in the control group was not specified. Blinding was not clearly described either and so there is an unclear risk of bias. Therefore, immunisation as an approach to prevent UTIs in pregnancy needs further exploration to assess its feasibility in practice.

### Ascorbic acid

Ochoa-Brust et al. [31] concluded that daily ascorbic acid was beneficial especially in areas with a high incidence of UTIs and antimicrobial resistance. This is a promising result but requires additional trials to strengthen the evidence before it can be recommended. It is not clear whether the authors used intention-to-treat analysis because they did not specify the withdrawal rate and there was a selection bias as they excluded women who were non-adherent or had ‘serious side effects’ from the medication. Excluding these results from analysis may distort the results in favour of ascorbic acid. It is worth noting, however, that no harmful effects were observed in the offspring of women who ingested ascorbic acid daily.

### Canephron® N

Canephron® N is a phytotherapeutic medicine with antibacterial properties and contains three herbs namely rosemary, lovage and centaury [38]. It is manufactured by a German company, Bionorica®, which focuses on researching and developing plant-based medicines. Ordzhonikidze et al. [32] conducted a study with pregnant women using this product, to optimise management of urinary tract diseases including ASB and pyelonephritis, which concluded that it could be recommended for prevention of urinary tract complications in pregnancy. The reporting of results was not comprehensive so it was not possible to determine how the study was conducted in sufficient detail (see Table 3). A review by Naber et al. [38] assessing the efficacy of Canephron® N suggests that there might be some benefit from its use in pregnant women because it included evidence from additional studies which have not been discussed here as they were conducted in pregnant women with co-morbidities and so did not meet the inclusion criteria of this review. It is worth noting that the safety of Canephron® N in pregnancy has been established [39, 40] but in order to make an evidence based recommendation, its efficacy needs to be confirmed by a randomised controlled trial.

## Strengths and limitations

A total of ten databases were searched and search terms were mutually agreed by the authors and an independent colleague to ensure a comprehensive process. The studies included in the review were assessed independently by the authors using CASP checklists. Any disagreement was resolved by meeting and discussing the relevant studies. A limitation of this review is that only English language publications were included therefore there might be options which have not been identified. The results of this review have been discussed using a narrative synthesis approach due to the heterogeneous design of the included studies and the differing nature of the interventions identified.

## Conclusion

All the approaches identified in this review are reported to be safe and effective. However apart from hygiene behaviours, the evidence behind these approaches is not robust enough to be recommended in practice. Future work needs to focus on strengthening the evidence base through improved design and reporting of clinical trials, in particular for the use of immunisation, ascorbic acid and Canephron® N. It is important that evidence based non-antibiotic measures to prevent UTIs in pregnancy are discovered to combat the danger that antimicrobial resistance poses to the health of this vulnerable patient group as well as the wider population.

## Additional file

Additional File 1: Search strategy. The additional file 1 contains the search strategy used to retrieve publications from the databases. It also contains details of authors who were contacted to obtain full text articles. (DOCX 16 kb)

## Abbreviations

ASB: Asymptomatic bacteriuria
Defra: Department for Environment Food and Rural Affairs
DH: Department of Health
EAU: European Association of Urology
RCT: Randomised control trial
SIGN: Scottish Intercollegiate Guidelines Network
UTI: Urinary tract infection
