# Supplementary material for: A systematic review of non-antibiotic measures for the prevention of urinary tract infections in pregnancy
Source: BMC Pregnancy Childbirth. 2018 Apr 13;18:99. doi: 10.1186/s12884-018-1732-2 (PMC5899369; doi:10.1186/s12884-018-1732-2)
Supplement: Supplementary file 1 — Search strategy. The additional file 1 contains the search strategy used to retrieve publications from the databases. It also contains details of authors who were contacted to obtain full text articles. (DOCX 16 kb) [file 12884_2018_1732_MOESM1_ESM.docx]

**Additional file – search strategy**

| **Database** | **Search terms** | **Records Retrieved** |
| --- | --- | --- |
| EMBASE | 1 "urinary tract infection" or UTI or bacteriuria or cystitis | 18,529 |
|  | 2 prevention or control or management | 1,041,064 |
|  | 3 pregnan* | 266,954 |
|  | 1 AND 2 AND 3  Limits: English language | **744** |
| AMED | 1 "urinary tract infection" OR UTI OR bacteriuria OR cystitis | 199 |
|  | 2 prevention OR control OR management | 37,458 |
|  | 3 pregnan* | 1,316 |
|  | 1 AND 2 AND 3  Limits: English language | **0** |
| BNI | 1 "urinary tract infection" OR UTI OR bacteriuria OR cystitis | 910 |
|  | 2 prevention OR control OR management | 101, 002 |
|  | 3 pregnan* | 18, 193 |
|  | 1 AND 2 AND 3 | **10** |
| CINAHL | 1 "urinary tract infection" OR UTI OR bacteriuria OR cystitis | 3,945 |
|  | 2 prevention OR control OR management | 443,963 |
|  | 3 pregnan* | 57,199 |
|  | 1 AND 2 AND 3  Limits: English language | **66** |
| MEDLINE | 1 "urinary tract infection" OR UTI OR bacteriuria OR cystitis | 34, 570 |
|  | 2 prevention OR control OR management | 3,251,851 |
|  | 3 pregnan* | 424,799 |
|  | 1 AND 2 AND 3  Limits: English language | **397** |
| PUBMED | 1 "urinary tract infection" OR UTI OR bacteriuria OR cystitis | 40,685 |
|  | 2 prevention OR control OR management | 5,532,953 |
|  | 3 pregnan* | 902,380 |
|  | 1 AND 2 AND 3 | **942** |
| PsycINFO | 1 "urinary tract infection" OR UTI OR bacteriuria OR cystitis | 574 |
|  | 2 prevention OR control OR management | 608,142 |
|  | 3 pregnan* | 39,650 |
|  | 1 AND 2 AND 3  Limits: English language | **4** |
| Cochrane | 1 "urinary tract infection" OR UTI OR bacteriuria OR cystitis | 6892 |
|  | 2 prevention OR control OR management | 372680 |
|  | 3 pregnan* |  |
|  | 1 AND 2 AND 3 (Trials) | **102** |
| Scopus | ( TITLE-ABS-KEY ( "urinary tract infection" OR UTI OR bacteriuria OR cystitis ) AND TITLE-ABS-KEY ( prevention or control or management ) AND TITLE-ABS-KEY ( pregnan* ) AND NOT TITLE-ABS-KEY ( catheter OR catheter AND associated ) AND NOT TITLE-ABS-KEY ( antibacterial* OR antibiotic* OR antimicrobial* )  Note: additional terms searched using ‘NOT’ due to too many results | **1008** |
| ScienceDirect  (searching abstract +title + keyword gave 7,234 results) | KEYWORDS ("urinary tract infection" OR uti OR bacteriuria OR cystitis) and KEYWORDS (prevention OR control OR management). | **3** |
| Manual search |  | **0** |

The authors of two systematic reviews were contacted to locate full text articles which they had included in their work but which were not available on open access:

- Ruth Jepson for conference abstract of study by Essadi et al (2010).
  - Jepson RG, Williams G, Craig JC. Cranberries for preventing urinary tract infections. Cochrane Database Syst Rev. 2012;10. doi: 10.1002/14651858.CD001321.pub5.
- Kurt Naber for full text of study by Ordzhonikidze et al (2009).
  - Naber KG. Efficacy and safety of the phytotherapeutic drug Canephron® N in prevention and treatment of urogenital and gestational disease: Review of clinical experience in Eastern Europe and Central Asia. Res Rep Urol. 2013;5:39–46. doi: 10.2147/RRU.S39288.
